# Impairment of regulatory T cell stability in axial spondyloarthritis: role of EZH2 and pSTAT5

**DOI:** 10.3389/fimmu.2024.1484321

**Published:** 2024-11-06

**Authors:** Majda Lyna Mebrek, Tessnime Abaab, Delphine Lemeiter, Magali Breckler, Roxane Hervé, Mylène Petit, Gaëlle Clavel, Johanna Sigaux, Marie-Christophe Boissier, Luca Semerano, Jérôme Biton, Natacha Bessis

**Affiliations:** ^1^ UMR 1125 INSERM, Bobigny, France; ^2^ Sorbonne Paris Nord University, Bobigny, France; ^3^ Assistance-Publique Hôpitaux de Paris (AP-HP), GHUPSSD, Department of Rheumatology, Bobigny, France; ^4^ Department of Internal Medicine, Fondation Rothschild, Paris, France

**Keywords:** regulatory T cells, spondyloarthritis, TNF inhibitors, EZH2, PSTAT5

## Abstract

**Background and objectives:**

Axial spondyloarthritis (axSpA) is a chronic inflammatory disease involving the spine, peripheral joints, and entheses. Functional impairment of regulatory T cells (Treg) is linked to inflammatory diseases, but limited data is available regarding Treg involvement in axSpA. Treg stability refers to their ability to maintain their functions and characteristics in pro-inflammatory environments. EZH2 and phosphorylated STAT5 (pSTAT5) play a critical role in maintaining Treg stability. We aimed to characterize Treg stability in patients with axSpA.

**Methods:**

Peripheral blood mononuclear cells (PBMCs) from axSpA patients, either naïve from targeted therapy or treated by TNF inhibitors (TNFi), and from healthy donors (HD), were freshly isolated. Expression of stability (EZH2, pSTAT5) and suppressive (TNFR2 and CD39) markers by Treg was analyzed by flow cytometry.

**Results:**

EZH2 expression by Treg was decreased in axSpA patients as compared to HD (p<0.01). Mechanistic study showed that inhibition of EZH2 attenuated Treg differentiation and suppressive phenotype *in vitro*. EZH2 was predominantly expressed by highly suppressive TNFR2^+^ and CD39^+^ Treg. Additionally, axSpA patients also exhibited a reduced frequency of pSTAT5 ^+^ Treg compared to HD (p<0.05), and pSTAT5 ^+^ Treg frequency increased at 3 months of TNFi treatment compared to baseline (p<0.05). This last result suggested a restoration of Treg stability upon TNFi treatment.

**Conclusion:**

By highlighting a deficient expression of EZH2 and pSTAT5 by Treg, we revealed an impaired Treg stability in axSpA. Deciphering the pathways influenced by these molecules is necessary to assess the potential therapeutic benefits of restoring Treg stability in axSpA.

## Introduction

Axial spondyloarthritis (axSpA) is an inflammatory disorder that affects the joints, entheses, and bone tissues and is sometimes associated with psoriasis, gut inflammation and anterior uveitis. Its pathogenesis is not fully understood and treatment strategies require optimization. Regulatory T cells (Treg) are pivotal in immune regulation, ensuring tolerance to self-antigens and preventing autoimmune reactions and excessive inflammation. Emerging evidence indicate that Treg imbalance or dysfunction could play a role in axSpA development (1). However, findings regarding the percentage of circulating Treg in axSpA patients yielded contradictory results (2), while researches focus on the study of their suppressive function are still limited. Given the pivotal role of Treg in autoimmune disorders (3), it is crucial to conduct further investigations into the role of Treg in axSpA.

In recent years, it has become increasingly clear that studying the phenotype and suppressive function of Treg are not sufficient to predict their immuno-modulatory action. There is growing evidence that Treg stability should also be considered (4). Actually, Treg stability refers to the ability of Treg to maintain their function and phenotype in pro-inflammatory surroundings. Indeed, chronic inflammatory conditions such as axSpA could alter the stability of Treg, leading to a shift towards pro-inflammatory CD4 ^+^ T cells (4).

Treg stability maintenance is governed by several factors, including Helios (5), EZH2 and the phosphorylated form of STAT5 (pSTAT5). Concerning the latter, its phosphorylation participates in Treg maintenance. Indeed, Treg are highly responsive to IL-2, and STAT5 phosphorylation downstream of the IL-2 receptor (IL-2R) promotes Foxp3 expression. Thus, STAT5 activation in response to IL-2 is critical for Treg stability and suppressive activity (6). EZH2 also plays a critical role in Treg stability. It is an enzyme responsible for histone methylation through the addition of methyl groups to histone H3 at lysine 27 (H3K27). EZH2 leads to the formation of heterochromatin and to the silencing of pro-inflammatory genes by Treg. Thus, by supporting the Foxp3-driven gene-expression program, EZH2 maintains Treg identity (7).

The objective of this study was to investigate and characterize the stability of Treg in patients with axSpA by examining the expression and role of EZH2 and pSTAT5.

## Methods

### Patients and healthy donors

The characteristics of the patients are presented in **Table 1**. Individuals meeting the ASAS criteria for axial spondyloarthritis were selected. Sixteen axSpA (cohort 1) patients naïve of targeted therapy were recruited to study EZH2, TNFR2, CD39 and Helios expression. Twenty-two axSpA patients (cohort 2) naïve of targeted therapy were recruited for pSTAT5 evaluation. We also recruited 11 patients (cohort 3) starting TNFi. According to EULAR recommendations, patients received TNFi if they had a BASDAI > 40 or if they needed daily treatment with NSAIDs independently of the BASDAI score. All axSpA patients were recruited as part of their medical management in the rheumatology department of Avicenne Hospital, Bobigny, France.

**Table 1:** Characteristic of patients with axSpA.

Parameters	axSpA patients Cohort 1	axSpA patients Cohort 2	axSpA patients Cohort 3	
Number	16	22	11	
Age	46.9 ± 11.7	44.9 ± 2.9	52.4 ± 3.7	
Female n (%)	9 (56.29%)	9 (40.9%)	6 (54%)	
Disease duration (years)	13.1 ± 3.3	8.6 ± 1.6	11.1 ± 2.3	
HLA-B27 positive n (%)	11 (73%) *	10 (59%)*	3 (37%)*	
			M0	M3
BASDAI (/100)	47.8 ± 11.9	46.4 ± 2.9	49.2 ± 4.0	44.1 ± 6.7
BASFI (/100)	48.2 ± 12.0	45.6 ± 4.7	49.1 ± 6.1	50.7 ± 11.4
CRP (mg/ml)	5.3 ± 1.3	5.5 ± 1.2	6.2 ± 2.6	4.0 ± 2.5
ESR (mm/1^st^ hour)	16.0 ± 4.0	16.8 ± 3.6	21.2 ± 8.7	20.3 ± 10.0
NSAID treatment n (%)	6 (37%)	7 (35%)	5 (45%)	2 (18%)
Oral Corticosteroid: Yes, n (%) Dosage (mg/day)	1 (6%) 5	0 (0%) NA	none	none
cDMARDS n (%)	Slz: 2 (12%) MTX: 1 (6%) None:13 (81%)	MTX: 1 (4%) None:21(96%)	MTX: 1 (9%) None: 10 (91%)	MTX: 1 (9%) None: 10 (91%)
Targeted therapy	none	none	adalimumab (n=6) infliximab (n=1) etanercept (n=3) certolizumab((n=1)	

**Figure 1:**
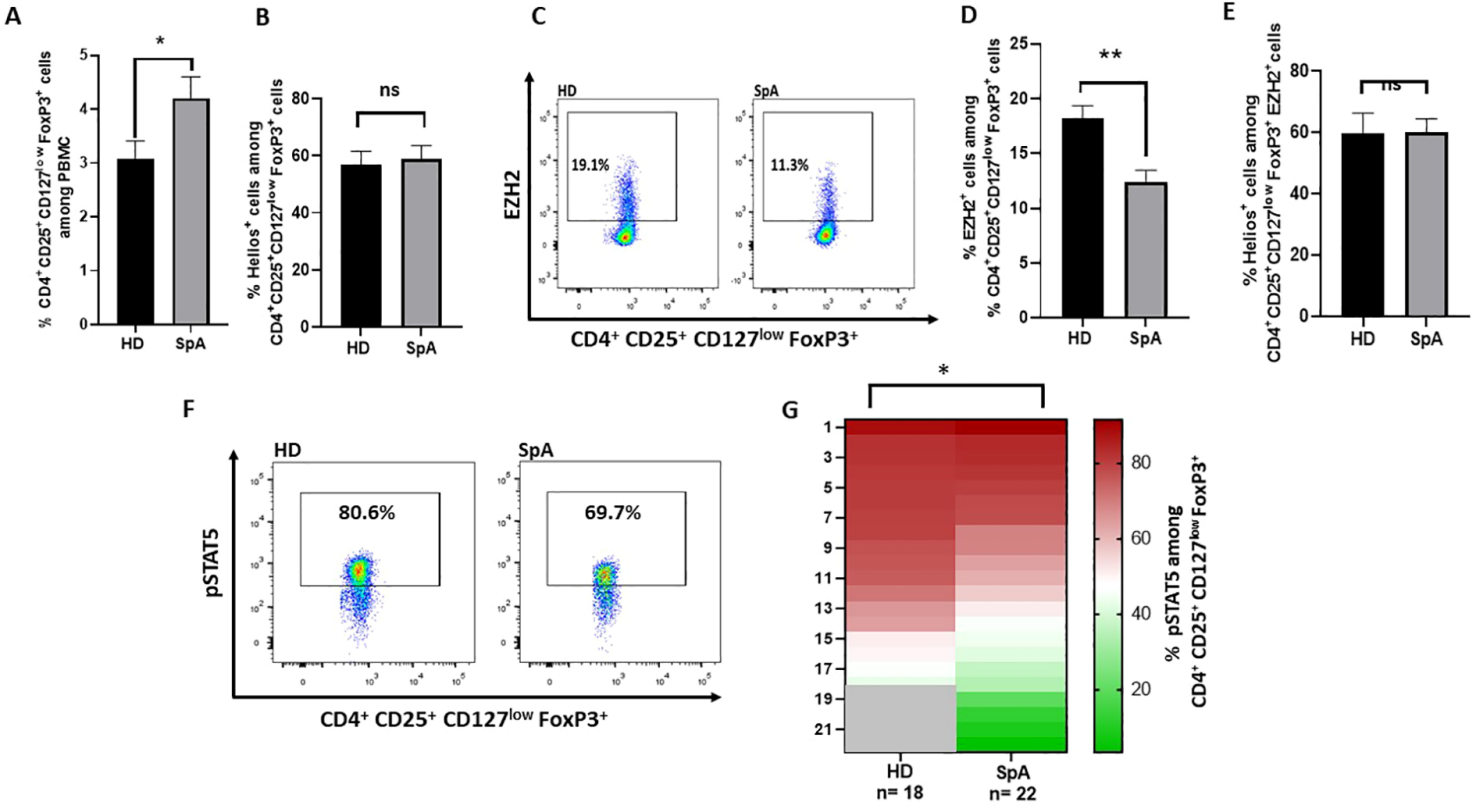
EZH2, pSTAT5 and Helios expression in Treg from axSpA patients and healthy donors (HD). **(A-F)** Flow cytometry experiments were performed using peripheral blood mononuclear cells (PBMC) from axSpA patients and from healthy donors (HD). **(A)** Frequency of Treg (CD4 ^+^ CD25 ^+^ CD127low FoxP3 ^+^ ) from axSpA patients (n=16) and HD (n=16). **(B)** Frequency of Helios ^+^ cells among Treg (CD4 ^+^ CD25 ^+^ CD127low FoxP3 ^+^ ) from axSpA patients (n=16) (cohort 1) and HD (n=16). **(C)** Representative dot plot of EZH2 expression among CD4 ^+^ CD25 ^+^ CD127^low^ FoxP3 ^+^ cells in axSpA patients and HD. **(D)** Frequency of EZH2 ^+^ cells among Treg (CD4 ^+^ CD25 ^+^ CD127^low^ FoxP3 ^+^ ) from axSpA patients (cohort 1) (n=16) and HD (n=17). **(E)** Frequency of Helios ^+^ cells among EZH2^+^ Treg (CD4 ^+^ CD25 ^+^ CD127^low^ FoxP3 ^+^ ) from axSpA patients (cohort 1) (n=16) and HD (n=17). **(F)** Representative dot plot of pSTAT5 ^+^ Treg (CD4 ^+^ CD25 ^+^ CD127^low^ FoxP3 ^+^) frequency among the indicated individuals. **(G)** Heatmap showing the frequency of pSTAT5 ^+^ cells among Treg (CD4 ^+^ CD25 ^+^ CD127^low^ FoxP3 ^+^ ) from axSpA patients naïve of targeted therapy (cohort 2) (n=22) and HD (n=18). Data are expressed as mean ± SEM. Unpaired t-test were used for statistical analysis. *p< 0.05; **p<0.01. ns, non significant.

**Figure 2:**
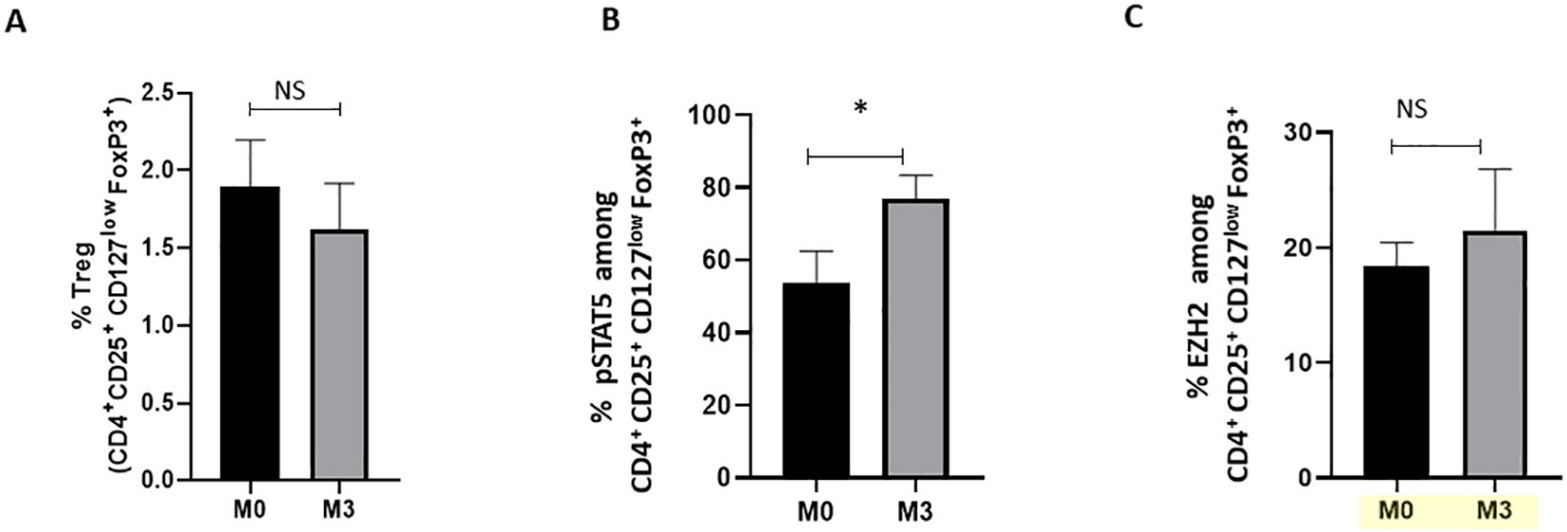
Impact of TNFi treatments on pSTAT5 and EZH2 expression in Treg from axSpA patients. Flow cytometry was used to analyse cells at baseline (M0) and 3 months (M3) after TNFi (adalimumab (n=6), infliximab (n=1), etanercept (n=3), certolizumab (n=1)) of axSpA patients (n=11) (cohort 3). **(A)** Percentage of CD4 ^+^ FoxP3 ^+^ Treg in peripheral blood. **(B, C)** Frequency of pSTAT5 ^+^ and EZH2 ^+^ cells among CD4 ^+^ CD25 ^+^ FoxP3 ^+^ CD127low Treg from axSpA patients at M0 and M3, respectively. Data are expressed as mean ± SEM. A Wilcoxon matched-pairs signed-rank test was used for statistical analysis. *p≤ 0.05. ns, non significant.

Mean ± SEM are shown. Cohorts 1 of SpA patients were used in **Figures 1A–E** and in **Supplementary Figures S2**, **S3**. Cohorts 2 of SpA patients were used in **Figures 1F, G**. Cohorts 3 of SpA patients were used in **Figure 2**. CRP, C-reactive protein; BASDAI, Bath Ankylosing Spondylitis Disease Activity Index; BASFI, Bath Ankylosing Spondylitis Functional Index; MTX, methotrexate; Slz, salazopyrine; NSAIDs, non-steroidal anti-inflammatory drugs; cDMARDs, conventional disease-modifying antirheumatic drugs; ESR, Erythrocyte Sedimentation Rate; NA, not applicable; M0, month 0; M3, 3 months after targeted therapy start.

* HLA B27 data not available for all patients.

Healthy donors (HD) were randomly selected and recruited from the « Etablissement Français du sang » (EFS) of Avicenne Hospital, Bobigny, France. The study was approved by the local ethics committee (NI-2016-11-01), and written informed consent was obtained from all patients before study entry.

### Mice

Male mice aged 6-12 weeks belonging to the C57BL/6 strain were purchased from Janvier (Le Genest-Saint-Isle, France). Homozygous TNFR2^-/-^ mice were generated as described in (8) by crossing wild-type (wt) C57BL/6 mice and double-deficient TNFR1/TNFR2 mice. All procedures were approved by the Animal Care Use Committee of the Sorbonne Paris Nord University (Bobigny, France) and the ethical committee Charles Darwin (#16312).

### Cell preparation

Peripheral blood mononuclear cells (PBMC) were freshly isolated by density gradient centrifugation (Polymorphprep, Proteogenix) from EDTA blood samples from axSpA patients and from healthy donors.

### Lymphocyte purification

Naive CD4 ^+^ CD44^-^ CD62L ^+^ T cells from mouse spleen were isolated using magnetic cell sorting (Naïve CD4 ^+^ T Cell isolation kit, mouse, Miltenyi Biotec) according to the manufacturer’s protocol. Flow cytometry analysis showed that the purity of the CD4 ^+^ CD44^-^ CD62L ^+^ cell-enriched fraction was > 90%.

### Flow cytometry

#### Patients and healthy donors

Surface markers staining for assessment of Treg from axSpA patients and from healthy donors, were carried with the following antibodies: BV510-labeled anti-CD4 (clone RPA.T4; BioLegend), PerCP-Cy5.5-labeled anti-CD25 (clone M-A251; BioLegend), APC-Cy7 -labeled anti-CD39 (clone M A1, BioLegend), PE-Cy7-labeled anti-CD127 (clone A019D5) and APC-labeled anti-TNFR2 (clone 3G7A02; BioLegend). Cells were stained at 4°C in PBS containing 5% heat-inactivated FCS, 0.02 M sodium azide and 100µg/mL human immunoglobulin (Merck Millipore), and incubated for 20 min with appropriate dilutions of various antibodies or corresponding isotype controls.

For intracellular Foxp3, EZH2 and Helios staining, cells were fixed and permeabilized with Foxp3 Transcription Factor Staining Buffer Set (Thermo Fisher Scientific). The staining was accomplished with the following antibodies: PE-labeled anti-Foxp3 (clone PCH 101; Thermo Fisher Scientific), BV-421-labeled anti-EZH2 (clone 11/EZH2, BD Bioscience) and AlexaFluor-488-labeled anti-Helios (clone: 22F6, Biolegend) and incubated for 30 min at 4°C in permeabilization buffer with appropriate dilution of various antibodies or corresponding isotype controls.

For pSTAT5 staining on Treg, PBMC were stimulated with recombinant human IL-2 (Miltenyi) for 15 minutes at 37°C, 5% CO2. Then, cells were fixed with pre-warmed Cytofix Buffer (BD Bioscience) at 37°C for 12 minutes, permeabilized with pre-chilled Perm Buffer III (Phosflow, BD Bioscience) for 30 minutes on ice, washed two times with PBS containing 5% FCS, and finally stained for 60 minutes at room temperature with BV510-labeled anti-CD4 (clone RPA.T4; BioLegend), eFluor450-labeled anti-FoxP3 (clone: PCH101, Thermo Fisher Scientific), and AlexaFluor488-labeled anti-STAT5 (pY694) (clone 47; BD Bioscience), or corresponding isotype control coupled to eFluor450 and AlexaFluor488 in PBS containing 5% FCS, 0.02 M sodium azide and 100μg/mL human immunoglobulin (Merck Millipore).

All flow cytometry data were acquired on a BD FACS CANTO II flow cytometer (Becton Dickinson). Gates were defined based on isotype controls and results were analyzed using FACS Diva software (BD Bioscience) and FlowJo software (BD Bioscience).

#### Mice

Cell surface markers were stained at 4°C in PBS containing 5% heat-inactivated FCS, 0.02 M sodium azide and 100µg/mL human immunoglobulin (Merck Millipore), and incubated for 20 min with appropriate dilutions of APC-Cy7-labeled anti-CD4 (clone RM4-5; BD Biosciences), PE-Cy7-labeled anti-CD39 (clone Duha59, Biolegend), biotin-labeled anti-TNFR2 (clone TR75-32.4, biolegend) and Biolegend Brilliant Violet 510™ Streptavidin or corresponding isotype controls.

For intra-nuclear Foxp3 and EZH2 staining, cells were first fixed and permeabilized with the eBioscience Foxp3 Transcription Factor Staining Buffer Kit (Thermo Fisher Scientific), then staining was performed with APC-labeled anti-Foxp3 (clone FJK.16s; Thermo Fisher Scientific) and with BV421-labeled anti-EZH2 (clone 11/EZH2; BD Biosciences) or their corresponding APC-coupled and BV421-coupled isotype controls. Cells were incubated for 30 min at 4°C in permeabilization buffer with the appropriate dilution.

For IFNγ intracellular cytokine staining, cells were stimulated for 4 h with PMA, ionomycin (Sigma-Aldrich, St. Louis, MO) and Brefeldin A (BD Bioscience). For surface staining, cells were incubated with FITC- labeled anti-CD4 (clone RM4-5) for 30 min at 4°C in the dark, then washed. The cells were then permeabilized using Fixation/Permeabilization solution and stained with eFluor450-labeled anti-FoxP3 (FJK-16s) and APC-labeled anti–IFNγ (XMG1.2) for 30 min at 4°C in the dark.

For pSTAT5 staining on Treg, splenic CD4^+^ T cells were purified (Miltenyi 130-104-454) and stimulated with recombinant mouse IL-2 (Miltenyi) (1ng/ml) for 15 minutes at 37°C, 5% CO2. Then, cells were fixed with pre-warmed Lyse/Fix Buffer (Phosflow, BD Biosciences) at 37°C for 12 minutes, washed with PBS containing 5% FCS, permeabilized with pre-chilled Perm Buffer III (Phosflow, BD Biosciences) for 30 minutes on ice, washed two additional times with PBS containing 5% FCS, and finally stained for 60 minutes at room temperature with APC-Cy7-labeled anti-CD4 (clone GK1.5; BD Biosciences), eFluor450-labeled anti-FoxP3 (clone FJK.16s, eBioscience), and AlexaFluor488-labeled anti-STAT5(pY694) (clone 47; BD Biosciences), or corresponding isotype control coupled to, eFluor450 or AlexaFluor488 in PBS containing 5% FCS, 0.02 M sodium azide and 100μg/mL human immunoglobulin (Merck Millipore).

### Mouse CD4+ T cell cultures

CD4^+^ T cells from mouse spleen were isolated using magnetic cell sorting (CD4+ T Cell Isolation Kit, mouse, Miltenyi Biotec, 130-104-454) according to the manufacturer’s protocol.

CD4^+^ T cells (1.105 cells per well) from wt or TNFR2^-/-^ mice were cultured in plates coated with anti-CD3 (2µg/mL) (BD, 553057, clone: 145-2C11) and anti-CD28 antibodies (1µg/mL) (BD, 553294, clone: 37.51) in the presence or absence of mouse IL-2 (25pg/mL) (Miltenyi Biotec, 130-120-332) for 72 hours, 37°C, 5% CO2.

### Treg differentiation

For mouse *in vitro* regulatory T cell differentiation, CD4^+^ CD44^-^ CD62L^+^ naive splenic T cells were isolated from C57BL/6 mice as described in **Supplementary Materials** Then the cells (2,5 x 10^5^) were differentiated in flat bottom 96-well plates in «CellXVivo Mouse Treg Cell Differentiation Kit» medium (R&D Systems, CDK007) and cultured for 5 days. In some conditions, CPI-1205 (7µM) (Adooq Bioscience A16357) or dimethyl sulfoxide (DMSO) were added to the culture.

### Enzyme-linked immunosorbent assay

Cell-free culture supernatants were collected at the end of the culture period. Then, according to the manufacturer’s instructions, ELISA kit (R&D systems, DY285) was used to measure IFNγ concentrations.

### Assessment of Treg suppressive effect on CD4+ cells

For T cell suppression assay, autologous PBMC from HD frozen during the Treg differentiation period were thawed and labeled with CellTrace Violet (CTV) (Thermo Fisher Scientific) for 20 minutes at 37°C at a final concentration of 2,5 µM. PBMC-CTV^+^ and autologous differentiated Treg were co-cultured (ratio 1/1) in RPMI 1640 with 10% FCS, 100 U/ml penicillin, 100 mg/ml streptomycin, 50 mM 2-ME, 1 M HEPES, 0,5 µg/ml coated anti-CD3 (clone OKT3; Invitrogen) and 1 µg/ml soluble anti-CD28 (clone CD28.2; BD Pharmingen) mAbs in U-bottom 96-well plates for 72h. After 3 days of culture, cells were stained with APC-labeled anti-CD4 (clone RPA-T4, BD Bioscience) surface antibody, and proliferation was subsequently analyzed by flow cytometry (CellTrace Violet–based proliferation in combination with CD4 staining). Data were analyzed using FACS Diva software. The percentage of suppression was calculated as follows: % suppression = [(CD4^+^ proliferation without iTreg - CD4^+^ proliferation with iTreg)/CD4^+^ proliferation without iTreg] x100.

### Statistics

According to data distribution, a parametric (paired t test, unpaired t test) or a nonparametric (Wilcoxon, Mann–Whitney) test was used to compare data across the two groups. Data distribution was determined using a Shapiro normality test.

For all tests, differences were considered statistically significant at p < 0.05. Data are shown as mean ± SEM. All analyses were performed using GraphPad Prism version 9.0 (Graphpad Software, San Diego, California USA).

## Results

### Decreased EZH2 and pSTAT5 expression in Treg from axSpA patients

To assess the stability of Treg, we evaluated the frequency of Helios ^+^ , EZH2 ^+^ and pSTAT5 ^+^ cells among Treg in peripheral blood from targeted therapy naive axSpA patients. Healthy donors (HD) were used as controls. First, Treg frequency was higher in axSpA patients than in HD (**Figure 1A**). Although Helios expression by Treg was not different in axSpA patients compared to HD (**Figure 1B**), the frequency of EZH2 ^+^ cells among Treg from axSpA patients was significantly lower than in HD (**Figures 1C, D**). The absolute number of Treg expressing EZH2 was also decreased in axSpA patients as compared to HD (**Supplementary Figure S1C**). Approximately 60% of EZH2^+^ Tregs expressed Helios either in HD and axSpa patients (**Figure 1E**). In addition to EZH2, STAT5 phophorylation plays a key role in the stability of Foxp3 expression (9). Importantly, we showed a decrease in the frequency of pSTAT5 ^+^ cells among Treg in axSpA patients compared to HD (**Figures 1F, G**). Overall, the lower expression of EZH2 and pSTAT5 by Treg in axSpA patients support an impaired Treg stability in this disease.

### Link between EZH2 and the suppressive markers TNFR2 and CD39 in Treg

Next, we aimed to assess the link between the stability and the suppressive function of Treg. Highly suppressive Treg were identified using two well-defined markers of Treg suppressive activity, meaning TNFR2 and/or CD39 (8, 10). First, we compared the expression of EZH2 in TNFR2-positive and TNFR2-negative Treg. The frequency of EZH2 ^+^ cells was significantly higher in the TNFR2 ^+^ Treg compared to TNFR2^-^ Treg both in HD (**Supplementary Figure S2A**) and axSpA patients (**Supplementary Figure S2B**). EZH2 ^+^ Treg frequency was also significantly higher in CD39 ^+^ Treg than in CD39^-^ Treg in HD and in axSpA patients (**Supplementary Figures S2C, D**). Thus, EZH2 expression is higher among the highly suppressive CD39 ^+^ or TNFR2 ^+^ Treg. Remarkably, the frequency of EZH2 expressing Treg among the CD39 ^+^ , TNFR2 ^+^ and TNFR2 ^+^ CD39 ^+^ Treg populations were lower in axSpA patients than in HD (**Supplementary Figures S2E–G**). Regarding pSTAT5, the method used to study its expression by flow cytometry included a methanol permeabilization step, which is only compatible with a limited number of fluorochromes. This prevented us from studying the link between pSTAT5 expression and that of TNFR2 and/or CD39.

Anyway, to further explore the link between EZH2 and TNFR2, we examined the expression of EZH2 in Treg derived from TNFR2-deficient mice. Our results demonstrated that Treg from TNFR2-deficient mice expressed EZH2 at a lower frequency than those from TNFR2 wild-type mice (**Supplementary Figures S3H, I**). Conversely, in HD and axSpA patients, we asked whether EZH2 expression conferred higher suppressive marker expression in Treg. We showed that the frequency and MFI of TNFR2 were significantly lower in EZH2^-^ Treg compared to EZH2 ^+^ Treg both in HD and axSpA patients (**Supplementary Figures S3A, C**). As for TNFR2, the frequency and MFI of CD39 expressing cells were significantly lower in EZH2^-^ Treg than in their EZH2 ^+^ counterparts (**Supplementary Figures S3B, D**). Taken together, these data highlighted the link between Treg stability and their suppressive function. Remarkably, they also underscored the altered stability of Treg supposed to display a highly suppressive phenotype in SpA.

### Inhibition of EZH2 attenuates *in vitro* iTreg differentiation, suppressive phenotype and function

Treg differentiation from naive CD4 ^+^ T cells requires the expression of Foxp3. To assess the role of EZH2 on the acquisition of Foxp3 expression, we evaluated the effect of a specific EZH2 inhibitor (CPI-1205) on Treg differentiation *in vitro*. Naive CD4 ^+^ T cells derived from mouse splenocytes were differentiated *in vitro* into induced Treg (iTreg) in the presence of CPI-1205 or its control (DMSO). CPI-1205 significantly reduced iTreg differentiation *in vitro* (**Supplementary Figure S4A**). Furthermore, we observed an increased IFNγ level in the supernatant of iTreg differentiated with CPI-1205 (**Supplementary Figure S4B**). In our *in vitro* experiments, naive T cells are not fully differentiated into iTreg after 5 days. Consequently, the increased IFNγ production induced by the EZH2 inhibitor could be related to the presence of cells not differentiated into iTreg rather than that of IFNγ producing iTreg. IFNγ intracellular staining on iTreg clearly showed an increased frequency of IFNγ ^+^ cells among iTreg in presence of CPI-1205 (**Supplementary Figure S4C**). Finally, we showed that EZH2 inhibition during Treg differentiation led to a decreased expression of the stability factor pSTAT5 (**Supplementary Figure S4D**), while TNFR2 and CD39 expression were not modified (**Supplementary Figures S4E, F**).

Interestingly, to examine the role of EZH2 on human Treg function, we performed a suppression assay using iTreg differentiated in the presence or absence of CPI-1205. After differentiation, cells were co-cultured with thawed autologous CTV-labeled PBMC for 72 hours at a iTreg/PBMC ratio of 1/1. We demonstrated that the inhibition of CD4^+^ T cell proliferation by iTreg is reduced when these cells were differentiated in the presence of CPI-1205 (**Supplementary Figure S5**).

### pSTAT5, but not EZH2, expression is restored by TNFi targeted therapies in axSpA patients

As TNF inhibitors (TNFi) have demonstrated the ability to modify Treg phenotype (7, 9, 10), we evaluated whether it impacts pSTAT5 ^+^ and EZH2 ^+^ Treg frequency in axSpA patients. First, our findings revealed no significant modification in the frequency of peripheral blood Treg among axSpA patients treated with TNFi after 3 months (**Figure 2A**). Neither BASDAI nor BASFI scores (**Table 1**) showed a correlation with Treg, pSTAT5^+^ Treg or EZH2^+^ Treg frequencies (data not shown). Remarkably, we observed an increase in the frequency of pSTAT5 ^+^ cells (**Figure 2B**), but not of EZH2 ^+^ cells (**Figure 2C**), among Treg following 3 months of treatment compared to baseline. Altogether, our study revealed that Treg stability is deficient in axSpA patients, but can be partially restored under TNF inhibitors treatment.

## Discussion

Our study describes a defect in the expression of Treg stability markers in axSpA patients. Indeed, we report a significant decreased frequency of EZH2 and pSTAT5 expressing cells among Treg in axSpA patients, as compared to HD. In a more global context, this work also includes data supporting a role for EZH2 in maintaining Treg differentiation and stability. In previous studies, we and others identified highly immunosuppressive Treg subpopulations expressing CD39 or TNFR2 (8, 10, 11). Here, we reveal a link between EZH2 expression and that of suppressive markers (CD39 and TNFR2) in Treg, suggesting that highly suppressive Treg also represent a more stable subpopulation. In axSpA, highly suppressive Treg (TNFR2 ^+^ , CD39 ^+^ or TNFR2 ^+^ CD39 ^+^ Treg) had a lower expression of EZH2… This suggests that even highly suppressive Treg display a less stable phenotype in axSpA patients. Finally, in axSpA patients, we show that TNFi restore pSTAT5 expression by Treg, suggesting an enhanced stability.

We sought to explore the underlying mechanism of EZH2-mediated Treg stability. Consistent with Goswami et al. (12), we show that CPI-1205, a pharmacological inhibitor of EZH2, impairs iTreg differentiation *in vitro* and modifies their suppressive phenotype and function. Furthermore, we showed that EZH2 inhibition by CPI-1205 during iTreg differentiation induces higher IFNγ expression by iTreg and lower frequency of pSTAT5 ^+^ iTreg. We thus highlight the importance of EZH2 in the maintenance of Treg identity and suppressive capacity.

We show an alteration of STAT5 phosphorylation in Treg from axSpA patients compared to HD. pSTAT5 binds to demethylated CNS2, inducing FoxP3 expression and maintaining its stability, which leads to a strong Treg suppressive activity. Our results are in concordance with the study of Guo et al. (13) showing that in SpA patients with active disease, peripheral blood Treg showed reduced effectiveness in utilizing IL-2 and displayed low levels of STAT5 phosphorylation. It would thus be interesting to study whether the T cells in axSpA patients produce sufficient IL-2. These results, including ours, suggest the presence of functional defects in Treg in axSpA. Interestingly, the lower frequency of pSTAT5^+^ Treg in axSpA patients compared to HD was not associated with a decrease in overall Treg frequency in our study. While STAT5 phosphorylation is known to directly induce Foxp3 transcription in Treg, numerous other factors also influence Foxp3 gene expression. In fact, Foxp3 expression is regulated by a complex network of transcriptional, epigenetic, and signaling pathways (14). Therefore, pSTAT5 is not the only factor involved in regulating Foxp3 expression, and in our study, there is likely a combination of other factors that ultimately contributed to the increased frequency of Treg in axSpA patients.

Our study shows that TNFi treatments were associated with a significant increase in the frequency of Treg expressing pSTAT5 in axSpA patients. This suggests that TNFi can restore Treg functionality and stability. This observation mirrors outcomes observed in rheumatoid arthritis (RA), which highlighted the necessity of IL-2/STAT5 signaling for the expansion of adalimumab-induced Treg. In contrast with pSTAT5, TNF inhibitors didn’t restore EZH2 expression in Treg in our study. Further studies are therefore required to more thoroughly evaluate the effect of TNF-targeted therapies on EZH2 expression by Treg, for example, by increasing the patient sample size, stratifying responders and non-responders, and assessing various timepoints after treatment initiation.

The limitations of this study include the use of peripheral blood samples, which may not fully reflect the Treg dynamics within the affected tissues. However, as recently discussed in the review by Rodolfi et al. (1), investigating Treg at inflammation sites in AxSpA is difficult due to the challenges in accessing affected organs for clinical and research purposes. As a result, the only site where Treg have been studied is the synovial fluid obtained during a peripheral arthritis flare. The study in question suggested an accumulation of suppressive Treg within inflamed joints in patients with spondyloarthritides (15).

Another limitation is that our study establishes a link between TNFR2 and EZH2 but it would have been relevant to underscore these results with functional tests. It would thus be interesting to demonstrate that EZH2^+^ Treg from TNFR2-deficient mice are less suppressive than those from wild-type mice. To achieve this, we would need to sort EZH2^+^ Treg to test their ability to inhibit the proliferation of effector T cells in culture. However, since EZH2 has intracellular expression, mice lacking EZH2 specifically in Treg or EZH2 fluorescent reported mice would be needed.

Eventually, additional limitation is that we mainly observed markers of stability and suppression in Treg in patients with axSpA, which doesn’t allow us to establish whether Treg dynamics influence amelioration of disease states, or whether we are only observing secondary changes of Treg dynamics by inflammation. However, it is likely that Treg are actively involved in the disease improvement process, as indicated by a study suggesting that low-dose interleukin-2, by expanding and activating Treg, indicates potential for the treatment of this disease (16).

It would also be relevant to explore Treg stability by assessing FoxP3 TSDR methylation or its suppressive activity. Interestingly, Guo et al. (13) has shown that, unlike Treg from HD, peripheral blood Treg from patients with active SpA did not effectively inhibit naïve CD4^+^ T cell proliferation. Furthermore, this study demonstrates that active SpA patients harbor Treg cells with higher CpG methylation levels in the CNS2 region of the foxp3 gene. This suggests an increased instability of FoxP3 in these patients.

Our study highlights the importance of EZH2 and pSTAT5 as significant factors in Treg stability in axSpA. Further research is needed to explore their intricate roles in the development and function of Treg. By elucidating the specific pathways influenced by these drivers of Treg stability, it will become conceivable to design innovative targeted therapies that will specifically modulate Treg function in diseases such as axSpA.

## Data Availability

The datasets presented in this article are not readily available because N/A. Requests to access the datasets should be directed to NB, natacha.bessis@univ-paris13.fr.
