# Supplementary material for: Impairment of regulatory T cell stability in axial spondyloarthritis: role of EZH2 and pSTAT5
Source: Front Immunol. 2024 Nov 6;15:1484321. doi: 10.3389/fimmu.2024.1484321 (PMC11576896; doi:10.3389/fimmu.2024.1484321)
Supplement: Supplementary file 1 [file DataSheet1.docx]

**
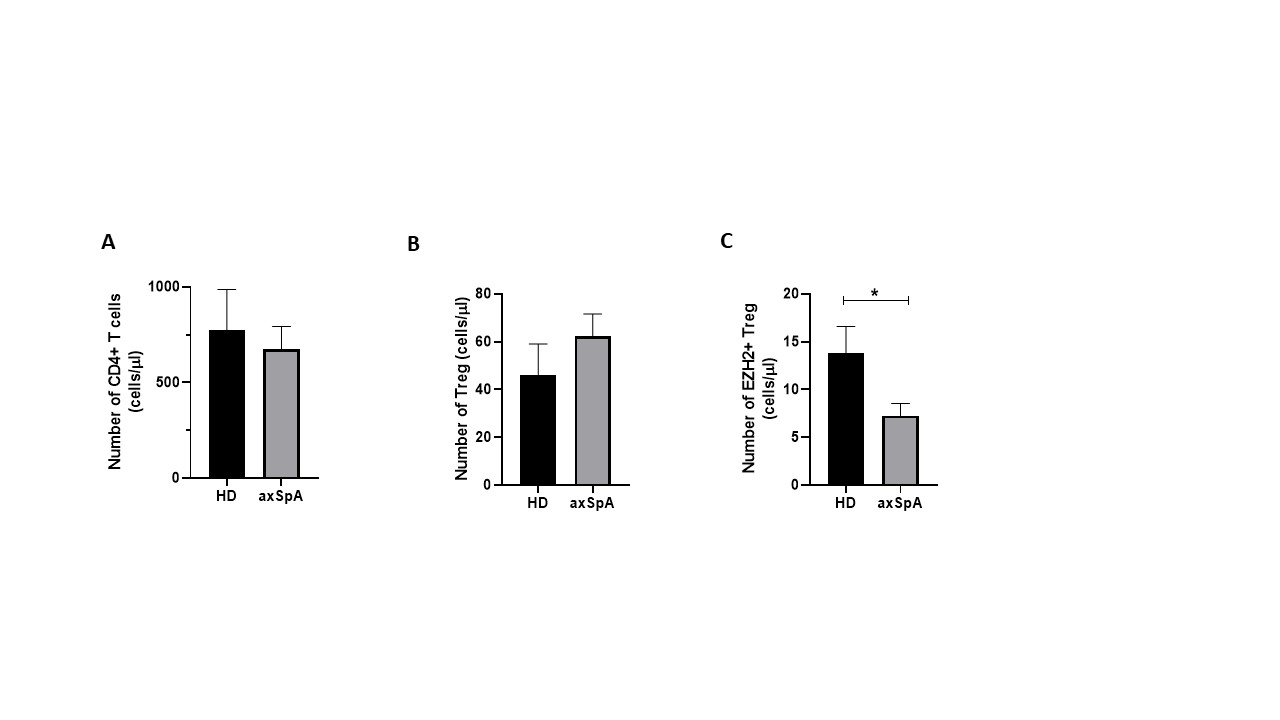
**

**Figure S1: Number of peripheral CD4^+^ T lymphocytes, Treg (CD4^+^ CD25^+^ CD127^low^ FoxP3^+^) and EZH2^+^ Treg (CD4^+^ CD25^+^ CD127^low^ FoxP3^+^ EZH2^+^) in axSpA patients and HD**. Flow cytometry experiments were performed using peripheral blood mononuclear cells (PBMC) from axSpA patients and from healthy donors (HD). axSpA patients and HD are the same as in figure 1D (cohort 1). (A) Number of CD4^+^ T lymphocytes per µl of PB. (B) Number of Treg (CD4^+^ CD25^+^ CD127^low^ FoxP3^+^) per µl of PB. (C) Number of EZH2^+^ Treg (CD4^+^ CD25^+^ CD127^low^ FoxP3^+^ EZH2^+^) per µl of PB. Data are expressed as mean ± SEM. Unpaired t-test was used for statistical analysis**.** * p< 0.05.

**
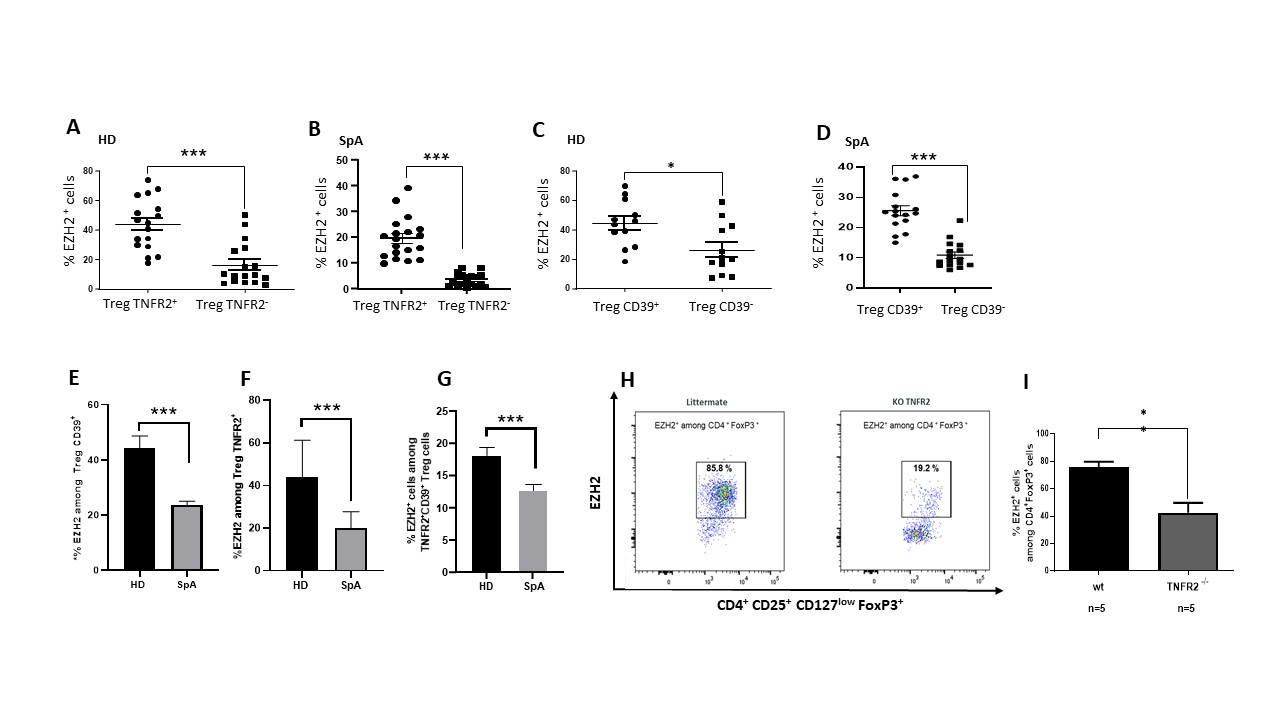
**

**Figure S2: Link between EZH2 and TNFR2 or CD39 suppressive markers.** Flow cytometry experiments were performed using peripheral blood mononuclear cells (PBMC) from SpA patients (cohort 1) and from healthy donors (HD). (A-D) Percentage of EZH2^+^ cells among Treg CD4^+^ CD25^+^ CD127^low^ FoxP3^+^ TNFR2^+^ or TNFR2 ^–^ (A, B) and CD39^+^ or CD39^–^ (C, D) from HD (A, C), and SpA (B, D) patients. (E-G) Frequency of EZH2^+^ cells among Treg (CD4^+^CD25^+^ CD127^low^ FoxP3^+^) CD39^+^ (E), TNFR2^+^ (F) and TNFR2^+^CD39^+^ (G) from SpA patients (n=16) and HD (n=17). (H) Representative dot plot of EZH2 expression by Treg and (I) frequency of EZH2^+^ cells among CD4^+^ FoxP3^+^ Treg in culture of CD4^+^ T cells from *wt* and TNFR2^-/-^ mice. Data are expressed as mean ± SEM. Unpaired t-test (A-G) and Mann-Whitney (I) were used for statistical analysis**.** * p< 0.02; ** p<0.01; *** p< 0.0001.

**
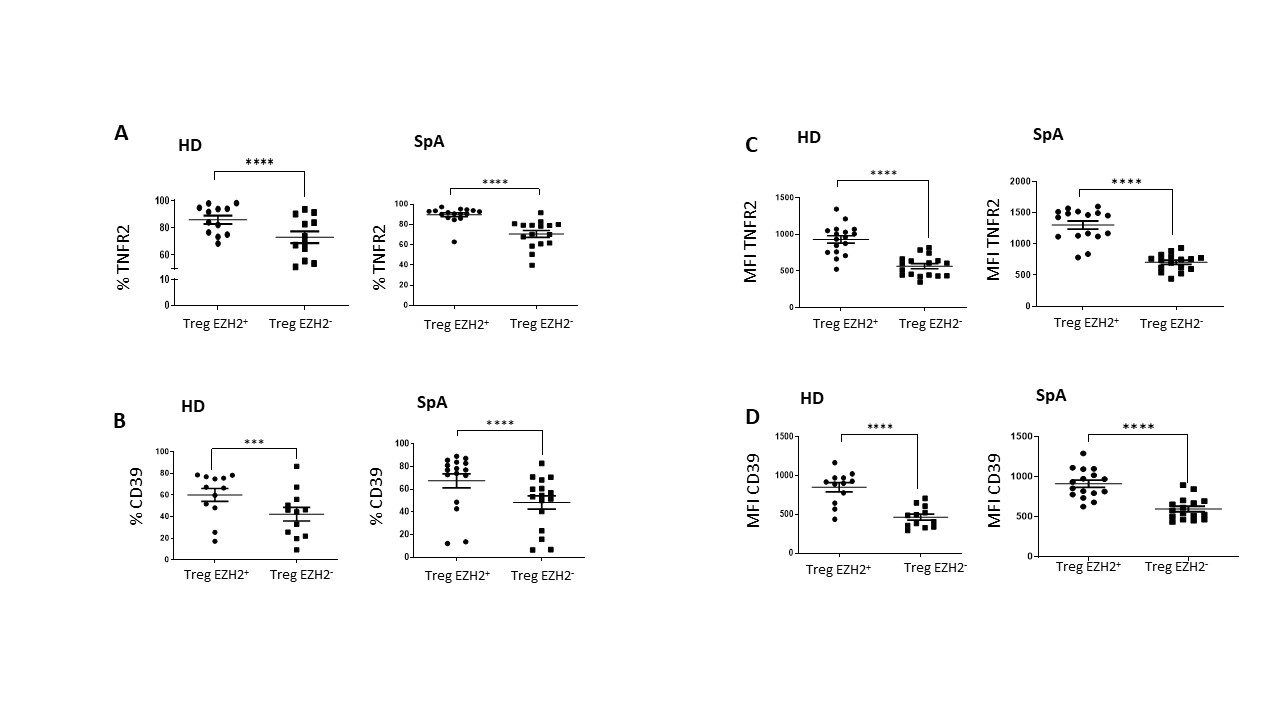
**

**Figure S3: Suppressive marker expression in EZH2^+^ or EZH2^-^ Treg populations.** Frequencies (A, B) and MFI (C, D) of TNFR2 and CD39 respectively among Treg EZH2^+^ or EZH2^-^ from HD and SpA patients (from cohort 1). All data were assessed by flow cytometry and are expressed as mean ± SEM. For statistical analysis, paired t-test was used. *** p< 0.001, **** p< 0.0001.


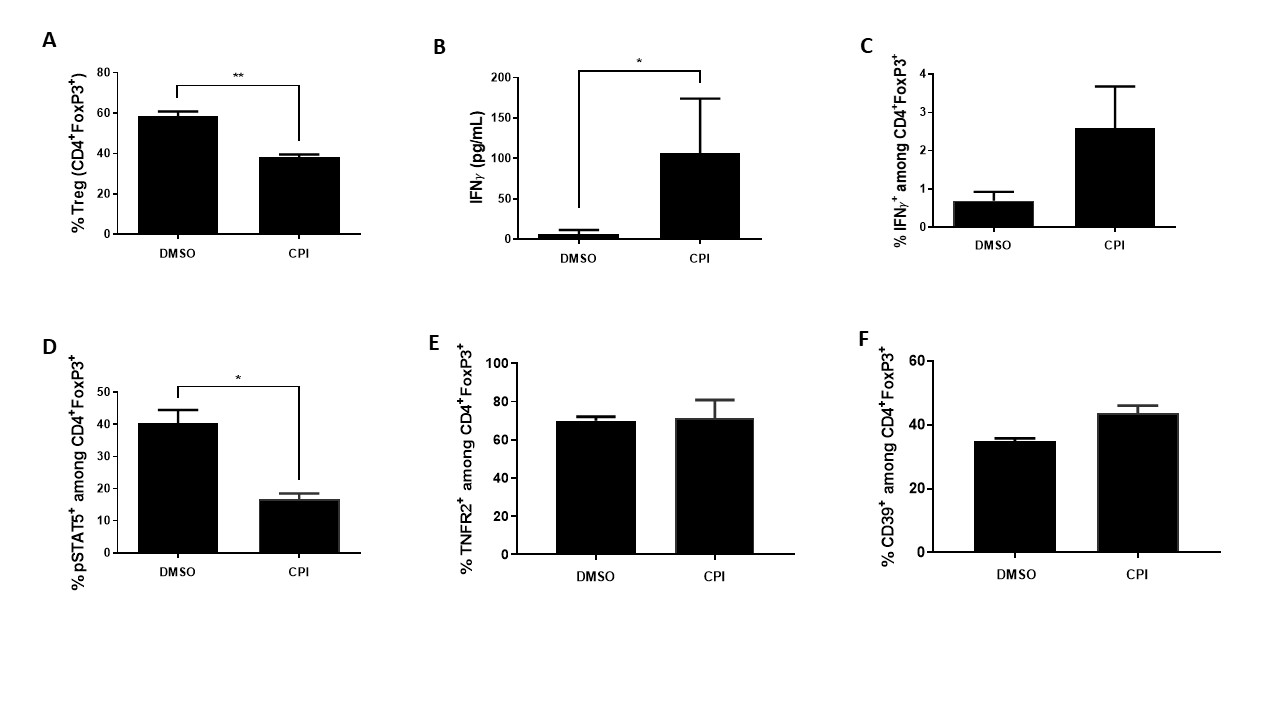


**Figure S4: EZH2 inhibition by CPI-1205 attenuates in vitro iTreg differentiation and suppressive phenotype.** Naive CD4^+^ T cells isolated from C57BL/6 mouse splenocytes were differentiated in vitro into iTreg in the presence of 7µM CPI-1205 or its control DMSO for 5 days. (A) CD4^+^ FoxP3^+^ Treg frequency among all cultured cells after 5 days. (B) Production of IFNγ in the culture supernatant (ELISA) of the iTreg differentiated for 5 days. (C) Frequency of IFNγ expressing cells among CD4^+^ FoxP3^+^ Treg assessed by intracellular staining after 5 days. Data are representative of 1 of 3 experiments, each yielding similar results. (D) Frequency of pSTAT5^+^ cells among CD4^+^ FoxP3^+^ Treg assessed by intracellular staining after 5 days (E) Frequency of TNFR2^+^ cells among CD4^+^ FoxP3^+^ Treg. (F) Frequency of CD39^+^ cells among CD4^+^ FoxP3^+^ Treg. Data are expressed as mean ± SEM. Mann-Whitney test was used for statistical analysis. ** p<0.01; * p< 0.05.


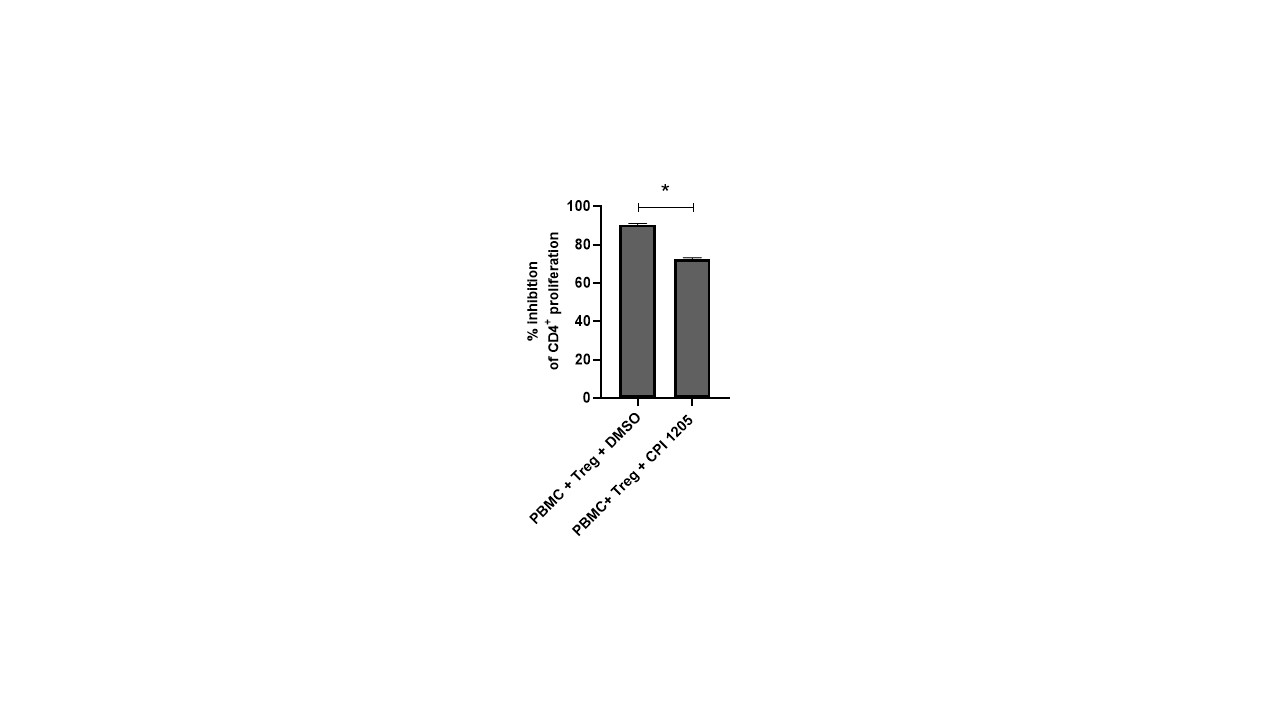


**Figure S5: EZH2 inhibition by CPI-1205 attenuates iTreg suppressive function.** Naive CD4^+^ T cells isolated from the peripheral blood of healthy donors were differentiated *in vitro* into iTreg in the presence of 7µM CPI-1205 or its control DMSO for 5 days The cells were then co-cultured with thawed autologous CellTrace Violet (CTV)-labeled PBMC for 72 hours at a ratio (1/1) with coated anti-CD3 antibodies (Abs) and soluble anti-CD28 Abs. The percentage of suppression was calculated as described in Materials and Methods. Data are expressed as mean ± SEM. Mann-Whitney test was used for statistical analysis. *p<0.05.
